# Effects of three probiotics and their interactions on the growth performance of and nutrient absorption in broilers

**DOI:** 10.7717/peerj.13308

**Published:** 2022-05-17

**Authors:** Lihuan Zhang, Yanfei Wang, Ruonan Zhang, Hao Jia, Xuan Liu, Zhiwei Zhu

**Affiliations:** Shanxi Agricultural University, Jinzhong, China

**Keywords:** Probiotics, Growth performance, Digestion and absorption, Intestinal morphology, GLUT2

## Abstract

The purpose of this study was to investigate the effects of three probiotics and their interactions on growth performance, intestinal digestion and absorption, and nutrient transporters in broilers. A total of 350 one-day-old male Arbor Acres broilers were randomly divided into seven groups: the control group (broilers receiving normal drinking water), groups P1, P2 and P3 (broilers receiving drinking water with 1% *Lactobacillus casei*, *Lactobacillus acidophilus* and *Bifidobacterium lactis* , respectively) and groups CP1, CP2 and CP3 (broilers receiving drinking water with a 1% compound probiotic mixture in 2:1:1, 1:2:1, 1:1:2 ratios, respectively). The feeding period was divided into two experimental periods: 1∼21 days and 22∼42 days. Compared to those in the control group, the broiler slaughter indexes and average daily feed intakes in the probiotics groups were not significantly different (*P* > 0.05), but the villus height in the small intestine increased significantly, and the crypt depth decreased significantly (*P* < 0.05). In the 1- to 21-day, experimental period, the broiler average daily gains in groups CP2 and CP3 were significantly greater than that in the control group. Amylase, lipase, and trypsin activities in the jejunum in groups CP and P3 increased significantly. GLUT2 mRNA expression in the probiotics group was significantly incresaed compared with that in the control group (*P* < 0.05). In the 22- to 42-day period, the average daily gain in the CP group was significantly greater than that in the control group. Amylase activity in the CP2 group, and lipase and trypsin activities in the CP, P1 and P3 groups increased significantly. The GLUT2 mRNA expression in the CP group increased significantly (*P* < 0.05). In summary, three probiotics and their interactions improved the digestibility and absorption of nutrients by increasing the activities of digestive enzymes, improving the morphology of the digestive tract, and upregulating the expression of GLUT2 mRNA in the intestinalcell membrane to improve the production performance in broilers.

## Introduction

In recent years, antibiotics have been widely used to meet the increasing demand for chicken meat. However, as a feed additive, antibiotics not only affects meat quality, but also potentially harm human health (Stanton, 2013). Previous studies have reported that antibiotic residues in chickens can enter the food chain and induce resistance in the consumer’s natural gut flora. An increase in drug-resistant bacteria can lead to gastrointestinal and nervous system diseases, and even death (Neogi et al., 2020; Neveling & Dicks, 2020). In recent years, researchers have found that probiotics have the potential to be used as safe and pollution-free feed additives.

Probiotics contain a mixture of one or more active microorganisms. When these microorganisms colonize the intestinal wall to a certain point, they can have a positive effect on the host (Alagawany et al., 2018). For example, probiotics can reduce the colonization rate of pathogenic bacteria on the intestinal wall, improve intestinal morphology, stimulate the immune system, improve metabolic function, and reduce the risk of infection. Their mode of action is a competitive rejection mechanism, as they compete with pathogenic bacteria in the intestinal tract for nutrients and prevent harmful bacteria from adhering to the intestinal epithelium (Caly et al., 2015; Bogucka et al., 2019; De Cesare et al., 2020). The synergistic effect of the intestinal microstructure and intestinal microbes can affect intestinal digestion and absorption and regulate body weight and feed intake. Such stimuli include changes in the environment and diet (Clemente et al., 2012; Shi et al., 2017). In general, animals consume probiotics through feed or drinking water supplementation or direct comsumption of the bacterial liquid. Probiotics can change the richness and diversity of beneficial bacteria and positively affect the composition of intestinal microbes (Conlon & Bird, 2015). There are a wide variety of probiotics; the most common species belong to the genera *Lactobacillus* and *Bifidobacterium* (Gaggìa, Mattarelli & Biavati, 2010). Studies have shown that *Lactobacillus* can retain micronutrients by promoting the secretion of phytase to promote intestinal metabolism (Mohammadi Ziarat et al., 2020). In addition, lactic acid and short-chain fatty acids (SCFAs) produced by *Lactobacillus* can activate intestinal epithelial cells and immune cells, protect the intestinal barrier and maintain gastrointestinal homeostasis (Iraporda et al., 2015).

Animal growth depends on the digestion and absorption of nutrients in the gastrointestinal tract. The jejunum secretes digestive enzymes that degrade high-molecular-weight carbohydrates into monosaccharides. More than 98% of glucose is absorbed by the jejunum and supplies the body’s energy needs (Chukwuma & Islam, 2015). These monosaccharides act on specific transporters in the intestine, such as glucose transporter 2 (GLUT2). As a glucose transporter with a high transport capacity that exists in a wide variety of tissues, GLUT2 responds quickly to increasing glucose concentrations and transports glucose from the intestinal lumen in a passive manner to balance glucose levels inside and outside the cell (Mueckler & Thorens, 2013; Wu et al., 2020). Thus, the expression of GLUT2 in the intestine may be a key factor determining digestion and absorption capacity in the body.

The effects of probiotics depends on the efficacy of a single strain or synergism among multiple strains. According to current research, probiotics in drinking water or feed can improve the growth performance of broilers, but few studies have focused on the intestinal digestion and absorption of probiotics and related nutrient transport mechanisms in broilers. Therefore, this study used young broiler chicks as test subjects to explore the effects of three probiotics and their interactions on broiler growth performance, intestinal digestion and absorption, and nutrient transport proteins to maximize poultry production efficiency while ensuring consumer safety.

## Materials & Methods

### Test materials

The proposed study protocol was approved by the Animal Care and Use Committee of Shanxi Agricultural University (registered number, SXAU-EAW-2021C0630). Broilers were euthanized by intraperitoneal injection of sodium pentobarbital (100–200 mg/kg). There were no surviving animals at the end of the experiment. The tested strains (*Lactobacillus casei*, *Lactobacillus acidophilus*, and *Bifidobacterium lactis*) were purchased from Shanghai Danisco Additives Co., Ltd. The strain numbers are LC-12, JYLA-16, JYBR-190, the density of viable probiotics was 1 ×10^10^CFU/g, respectively. Each individual probiotic powder and the compound probiotic powders (each probiotic powder was mixed in proportions of 2:1:1, 1:2:1, and 1:1:2) were fermented in sterile milk at 37 °C for 14 h to produce single probiotic- or compound probiotic-fermented milk. The density of viable probiotics was 3 ×10^9^ CFU/mL.

### Experimental design and animal feeding

In this experiment, 350 1-day-old male Arbor Acres broilers were selected. Individuals were weighed and randomly divided into 7 groups with 5 replicates in each group and 10 chickens in each replicate, and there was no significant difference in mean body weight among the groups. The groups included the control group (broilers receiving normal drinking water), groups P1, P2 and P3 (broilers receiving drinking water with 1% *Lactobacillus casei*, *Lactobacillus acidophilus* and *Bifidobacterium lactis*, respectively) and groups CP1, CP2 and CP3 (broilers receiving drinking water with a 1% compound probiotic mixture a 2:1:1, 1:2:1, or 1:1:2 ratio, respectively). Probiotics group included P1, P2, P3, CP1, CP2 and CP3 groups. All groups were fed a basic diet: the nutritional components are shown in Table 1. The feeding period was divided into two stages: 1∼21 days and 22∼42 days. The humane endpoints of the study included Weight loss: weight loss of 20% to 25% of the animal’s original body weight, or symptoms of cachexia or wasting. Loss of appetite: loss of appetite for 5 days or poor appetite (50% less than 1000 normal food intake) for 7 days. Weak or dying: Unable to eat or drink. The animal is unable or barely able to stand for up to 24 h without anaesthesia or sedation, or exhibits lethargy with hypothermia. Etc.

**Table 1:** Composition of and nutrient levels in the basic diet.

Item	1∼21 days	22∼42 days
Diet composition (%)		
Corn	56.49	61.42
Soybean oil	2.22	3.00
Soybean meal	30.24	25.30
Cotton seed meal	5.00	5.00
Fishmeal	2.43	1.98
CaHCO_3_	1.60	1.39
Limestone	1.16	1.10
Methionine	0.15	0.05
NaCl	0.30	0.35
Choline	0.19	0.19
Premix^a^	0.22	0.22
Nutrient^b^(%)		
M (MJ kg^−1^)	12.12	12.54
Crude protein	21.00	19.00
Lysine	1.12	0.98
Methionine+	0.84	0.68
Cystine		
Calcium	1.00	0.90
Available phosphorus	0.30	0.30

**Notes.**

a The premix contained 0.2% trace elements and provided the following nutrients per kg feed: Fe 80 mg, Mn 80 mg, Zn 80 mg, I 0.35 mg, Se 0.15 mg. The premix contained 0.02% vitamins per kg feed: Vitamin D3 3000.00 IU, Vitamin E 30.00 IU, Vitamin K3 1.00 mg, Vitamin B1 2.00 mg, Vitamin B2 6.00 mg, Pantothenic acid 9.00 mg, Pyridoxine 5.00 mg, Niacin 30.00 mg, Vitamin B12 0.01 mg, Biotin 0.10 mg, Folic acid 0.30 mg.

b Nutrition level is a calculated value.

Broilers were housed in three-layer vertical cages and were vaccinated according to regulations. Feed was provided at 8:00 am and 8:00 pm every day, and the drinking water was changed every 8 h. The chicken house was disinfected regularly. Continuous light was provided for 23 h, with a dark interval of 1 h, to ensure broilers fed and drank freely. The temperature was maintained at 35 °C during the first week of brooding and then was gradually decreased to 23 °C through the end of the experiment. The food intake and health status of broilers were recorded daily.

### Determination of growth performance and slaughter performance

At the end of the feeding period, the broilers in each group were weighed. The broilers were prevented from eating for 10 h but could drink freely before weighing. The growth data were recorded, and the average daily gain (ADG), average daily feed intake (ADFI) and feed conversion rate (FCR =F/G) of the broilers in each group were calculated. Twenty broilers from each group were randomly slaughtered, and the slaughter indexes were calculated according to NY/T 823-2004 “Performance ferms and measurement for poultry” (The Ministry of Agriculture of the People’s Republic of China, 2004).

### Intestinal sampling and morphological measurement

The slaughtered broilers were dissected, and then the small intestine (duodenum, jejunum and ileum) was cut and rinsed with precooled normal saline. A 1-cm^2^ sample of each intestinal tissue was cut and put in 4% formaldehyde fixative overnight. After dehydration with ethanol and clearing with xylene, the intestinal segment was embedded in paraffin, sectioned at a thickness of 6 µm, stained with hematoxylin and eosin, and sealed with resin for observation. The morphological parameters (*e.g.*, villus height (VH), crypt depth (CD), and the ratio of villus height to crypt depth (V/C)) of each small intestine segment were determined. The jejunum tissues were cut and tied with a cotton thread at both incision ends and then stored in liquid nitrogen for subsequent analysis.

### Immunohistochemistry

The 6-µm thick jejunum tissue sections were treated with ethanol and xylene, sealed with 3% H_2_O_2_ for 15 min, and then treated with a blocking solution (18 µl Triton X-100, 5.7 mL PBS, 0.3 g BSA mixture) to block heterologous antigens; sections were held at 37 °C for 10 min to inhibit nonspecific staining. Tissue sections were incubated with diluted (1:300) rabbit-derived primary antibody against GLUT2 receptor (bs-0351r; Bioss) at 4 °C overnight. After washing with PBS, the tissue sections were stored in 1:200-diluted hrP-labeled anti-rabbit secondary antibody (GB23303; Servicebio) for 1 h. Then, the tissue sections were washed, incubated in DAB for 1 min, stained with hematoxylin nuclei for 20 s, and again treated with ethanol and xylene. The slices were sealed for microscopy. For each slice, three visual fields under 200 × magnification were randomly selected to be photographed under the same background light.

### Determination of digestive enzyme activity in the jejunum

Two grams of preprocessed jejunum contents were weighed and added to 18 ml of PBS (pH 7.4) to make a 10% homogenate. Then the samples were centrifuged at 3000 g for 20 min at low temperature, and the supernatant was collected. The activities of amylase, lipase and trypsin were measured according to the manufacturer’s instructions (Jiancheng Bioengineering Institute, Nanjing, China).

### Extraction of total RNA from the jejunum and qRT-PCR

Total RNA was extracted from the jejunum by the TRIzol method, and the concentration of RNA was detected by a NanoDrop 2000 (Thermo Fisher; M584). cDNA was obtained by using a reverse transcription kit (RR036A; Takara). The relative expression level of GLUT2 mRNA in the jejunum was determined by qRT-PCR with SYBR Green (Takara, SYBR Premix Ex Taq^TMII^ Kit). The primer sequences are provided in Table 2. The optimized PCR protocols consisted of initial denaturation at 95 °C for 5 min, followed by 40 cycles of denaturation at 95 °C for 5 s, 60 °C for 35 s, and 72 °C for 20 s. Each sample was measured in triplicate. The melting curve was analyzed to determine the specificity of the reaction. The *β*-actin gene was used as an internal control, and the relative expression of GLUT2 mRNA was calculated according to the formula: P =2^−ΔΔCT^.

### Statistical analysis of data

After preliminary arrangement of the data in Microsoft Excel 2018, ANOVA was performed, and Duncan’s test was used to determine differences among treatments in SPSS 24.0 statistical software. The histogram was built by GraphPad Prism 8.

## Results

### Growth performance and slaughter performance

The effects of the probiotics on the growth performance of broilers are shown in Table 3. At 1 to 21 days, the ADFIs and FCRs of broilers in the probiotics groups were not significantly different from those in the control group (*P* > 0.05), but the ADGs of broilers in groups CP2 and CP3 increased significantly (*P* < 0.05). Among the probiotics groups, the ADGs in groups CP2 and CP3 were significantly higher than those in groups P1 and P2 (*P* < 0.05). At 22 to 42 days, the FCR of broilers in group CP2 was significantly lower than that in the control group (*P* < 0.05). Compared to the control group, the ADG in the CP group increased significantly (*P* < 0.05), while that in the P group did not (*P* > 0.05). During the entire feeding period, the compound probiotics had more prominent effects on broiler growth performance than the single probiotics.

**Table 2:** Primers for real-time quantitative PCR.

Gene	Primer sequence (5′–3′)	DNA fragment (bp)	Anneal (°C)
*β*-actin	F:GTCCACCGCAAATGCTTCTA R:AGCCATGCCAATCTCCGTCTT	154	60
GLUT2	F:CCGTCCTCCTCCTGGTCTTCTTC R:AGCTTCTTGCGGCGGAATGC	103	60

**Table 3:** Effects of probiotics in water and feed on the growth performance of broilers. Different superscript letters in the same row indicate statistically significant differences (*P* < 0.05), This also applied to the table below.

Parameter	CON	Drinking water with probiotics						SEM	*P*-value
		CP1	CP2	CP3	P1	P2	P3		
1∼21 days									
ADG (g/bird)	20.95^b^	21.97^ab^	22.68^a^	22.73^a^	21.01^b^	21.02^b^	21.79^ab^	0.76	0.01
ADFI (g/bird)	40.85	40.49	40.84	41.53	40.45	40.84	40.61	1.94	0.63
FCR (g/g)	1.96	1.84	1.80	1.83	1.92	1.88	1.86	0.11	0.21
22∼42 days									
ADG (g/bird)	48.82^b^	53.89^a^	52.44^a^	52.60^a^	50.47^ab^	49.12^ab^	52.01^ab^	1.54	0.03
ADFI (g/bird)	98.93	100.12	99.32	100.35	100.68	99.27	103.29	2.85	0.19
FCR (g/g)	2.03^a^	1.91^ab^	1.85^b^	1.91^ab^	1.99^ab^	1.97^ab^	1.99^ab^	0.07	0.01

The effects of probiotics on the slaughter performance of broilers are shown in Table 4. Compared to that in the control group, the slaughter performance of broilers in the probiotics groups were not significantly different at 1 to 42 days (*P* > 0.05). However, among the probiotics groups at 22 to 42 days, the eviscerated carcass yield of broilers in group CP3 was significantly higher than those in groups CP1, P1 and P3, and the muscle stomach indexes in groups CP1 and CP3 were significantly higher than those in groups P1 and P3 (*P* < 0.05). Further, we found that the eviscerated carcass yield and the half-eviscerated carcass yield of all the test broilers were over 60% and 75%, respectively. The broilers in group CP3 had better slaughter performance than the broilers in the other groups.

**Table 4:** Effects of probiotics in water and feed on the slaughter performance of broilers (%).

Parameter	CON	Drinking water with probiotics						SEM	*P*-value
		CP1	CP2	CP3	P1	P2	P3		
1∼21 days									
Eviscerated carcass yield	62.43	64.25	68.78	66.57	65.49	69.23	70.38	0.09	0.42
Half-eviscerated carcass yield	80.83	80.30	80.35	78.94	82.27	77.53	79.05	0.04	0.33
Breast yield	13.73	13.23	13.13	13.87	13.37	11.98	12.63	0.02	0.45
Thigh yield	20.47	20.02	18.68	19.25	20.02	17.17	17.80	0.03	0.34
Carcass lean percentage	34.20	33.25	31.81	33.12	33.39	29.15	30.43	0.05	0.37
Abdominal fat yield	4.98	4.75	5.49	5.12	4.33	5.21	4.70	0.01	0.42
Pancreas index	0.36	0.40	0.42	0.40	0.32	0.31	0.32	0.06	0.11
Gland stomach index	0.59	0.51	0.53	0.55	0.54	0.55	0.54	0.06	0.25
Muscular stomach index	2.36	2.12	2.15	2.02	2.17	2.30	2.38	0.33	0.34
22∼42 days									
Eviscerated carcass yield	68.05^ab^	64.75^b^	67.10^ab^	68.82^a^	64.81^b^	65.99^ab^	64.91^b^	0.02	0.03
Half-eviscerated carcass yield	83.44	81.19	79.25	83.07	79.93	80.49	79.09	0.03	0.15
Breast yield	15.07	14.86	14.91	15.56	15.56	15.07	17.69	0.02	0.30
Thigh yield	20.54	20.19	20.48	19.86	19.84	21.16	19.30	0.01	0.08
Carcass lean percentage	37.61	35.06	35.39	35.43	35.39	36.22	36.99	0.03	0.37
Abdominal fat yield	5.73	7.16	7.41	5.56	5.61	5.03	5.65	0.02	0.23
Pancreas index	0.25	0.29	0.26	0.25	0.29	026	0.20	0.05	0.10
Gland stomach index	0.30	0.39	0.37	0.32	0.35	0.36	0.35	0.05	0.11
Muscular stomach index	1.82^ab^	2.19^a^	1.90^ab^	2.19^a^	1.74^b^	1.85^ab^	1.71^b^	0.17	0.04

### Activity of jejunal digestive enzymes

**Figure 1:**
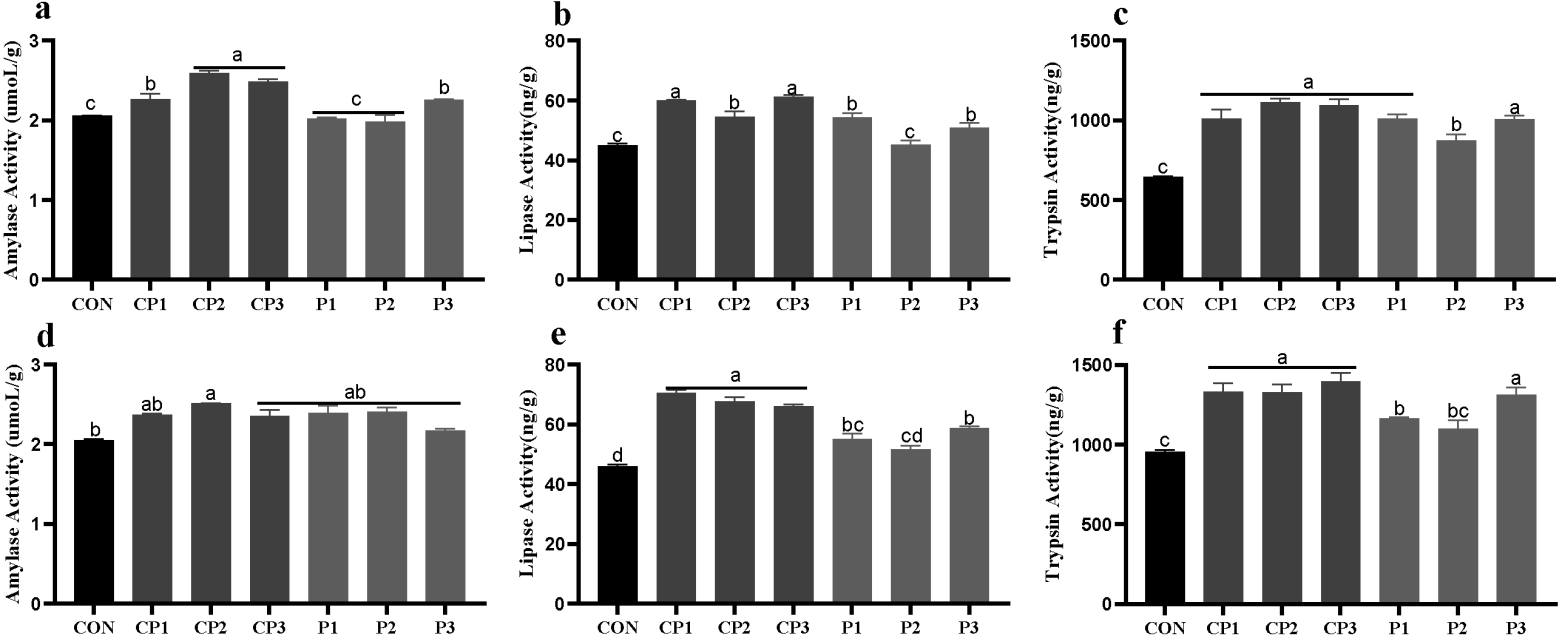
Effect of probiotics on jejunum amylase (AMY), lipase (LPS) and trypsin (TPS) activities at 1 to 21 days (A, B, and C) and 22 to 42 days (D, E, and F). The results are expressed as means ± standard deviations (M ± SD). Different letters above columns indicate statistically significant differences (*P* < 0.05). This also applied to the following figure. The control group (broilers receiving normal drinking water), groups P1, P2 and P3 (broilers receiving drinking water with 1% *Lactobacillus casei*, *Lactobacillus acidophilus* and *Bifidobacterium lactis*, respectively) and groups CP1, CP2 and CP3 (broilers receiving drinking water with a 1% compound probiotic mixture in 2:1:1, 1:2:1, 1:1:2 ratios, respectively).

At 1 to 21 days, AMY (amylase), LPS (lipase)and TPS (trypsin) activities in groups CP and P3 were significantly higher than those in the control group; the LPS and TPS activities in group P1 were increased significantly; and the TPS activity in group P2 was increased significantly (*P* < 0.05). Among the probiotics groups, AMY activity was the highest in groups CP2 and CP3, LPS activity was the highest in groups CP1 and CP3, and TPS activity in group P2 was significantly lower than those in the other groups (*P* < 0.05, Figs. 1A–1C). At 22 to 42 days, the AMY activity in group CP2 was significantly increased compared with that in the control group (*P* < 0.05), but AMY activity in the other probiotics groups was not significantly increased (*P* > 0.05, Fig. 1D). LPS and TPS activities in the probiotics groups, except for group P2, were increased significantly (*P* < 0.05). Among the probiotics group, LPS and TPS activities in the CP group were significantly higher than those in the P1 and P2 groups (*P* < 0.05, Figs. 1E–1F).

### Morphology of the small intestine

The effects of probiotics on the morphology of the small intestine are shown in Table 5. Compared to those in the control group, the VH and V/C ratio in the small intestine (duodenum, jejunum and ileum) in the probiotics group increased significantly, while the CD decreased significantly (*P* < 0.05). At 1 to 21 days, the jejunum in group CP2, and the duodenum and ileum in group CP3 had the highest VHs, and the jejunum in group CP3 had the lowest CD and the highest V/C ratio (*P* < 0.05). At 22 to 42 days, the duodenum and jejunum in group CP1 and the ileum in group CP2 had the highest VHs and V/C ratios, and the jejunum and ileum in group CP1, and the duodenum in group CP2 had the lowest CDs (*P* < 0.05). Compared probiotics had better effects on small intestine morphology than single probiotics.

**Table 5:** Effect of probiotics on morphological changes in the small intestine.

Parameter	CON	Drinking water with probiotics						SEM	*P*-value
		CP1	CP2	CP3	P1	P2	P3		
1∼21 days									
**Duodenum**									
Villus height/µm	1578.8^e^	1798.1^b^	1779.4^c^	1852.5^a^	1698.7^d^	1702.0^d^	1781.2^c^	18.88	0.01
Crypt depth/µm	225.5^a^	203.1^c^	189.4^d^	187.9^d^	208.9^b^	209.8^b^	209.4^b^	13.08	0.01
V/C ratio	6.9^d^	8.8^b^	9.4^a^	9.8^a^	8.0^c^	8.1^c^	8.5^c^	0.53	0.01
**Jejunum**									
Villus height/µm	1230.3^f^	1482.4^c^	1648.9^a^	1608.3^b^	1292.3^e^	1494.0^c^	1391.6^d^	17.08	0.01
Crypt depth/µm	188.3^a^	161.9^c^	169.6^c^	152.2^d^	173.5^b^	174.9^b^	173.4^b^	9.36	0.02
V/C ratio	6.5^e^	9.1^b^	9.7^b^	10.5^a^	7.4^d^	8.5^c^	8.0^c^	0.59	0.01
**Ileum**									
Villus height/µm	1081.8^f^	1258.8^b^	1154.3^c^	1363.7^a^	1100.9^e^	1104.3^e^	1134.7^d^	21.21	0.01
Crypt depth/µm	133.7^a^	130.5^b^	130.0^b^	130.9^b^	129.7^b^	129.3^b^	129.4^b^	12.00	0.02
V/C ratio	8.1^e^	9.6^b^	8.8^c^	10.4^a^	8.5^d^	8.5^d^	8.8^c^	0.83	0.01
22∼42 days									
**Duodenum**									
Villus height/µm	1478.1^e^	1954.8^a^	1576.4^b^	1512.2^c^	1495.5^d^	1502.3^d^	1502.9^d^	11.95	0.01
Crypt depth/µm	195.3^a^	172.4^d^	146.5^e^	170.5^d^	187.6^c^	191.6^b^	172.9^d^	11.13	0.01
V/C ratio	7.6^e^	11.3^a^	10.8^b^	8.9^c^	8.0^d^	7.8^d^	8.7^c^	0.61	0.01
**Jejunum**									
Villus height/µm	1220.1^e^	1367.1^a^	1262.8^c^	1285.7^b^	1259.7^c^	1237.9^d^	1239.9^d^	27.27	0.01
Crypt depth/µm	205.6^a^	132.5^d^	162.0^c^	163.1^c^	163.1^c^	190.3^b^	160.8^c^	14.85	0.01
V/C ratio	5.9^d^	10.3^a^	7.8^b^	7.9^b^	7.7^b^	6.5^c^	7.6^b^	0.56	0.02
**Ileum**									
Villus height/µm	832.5^e^	1072.6^b^	1325.2^a^	1076.5^b^	966.2^c^	955.1^c^	893.6^d^	23.35	0.01
Crypt depth/µm	145.3^a^	131.1^d^	138.8 ^bc^	137.4^c^	140.3^b^	140.4^b^	140.1^b^	11.19	0.03
V/C ratio	5.7^d^	8.2^b^	9.5^a^	7.8^b^	6.9^c^	6.8^c^	6.4^c^	0.65	0.01

### Localization and expression of GLUT2 in the jejunum

**Figure 2:**
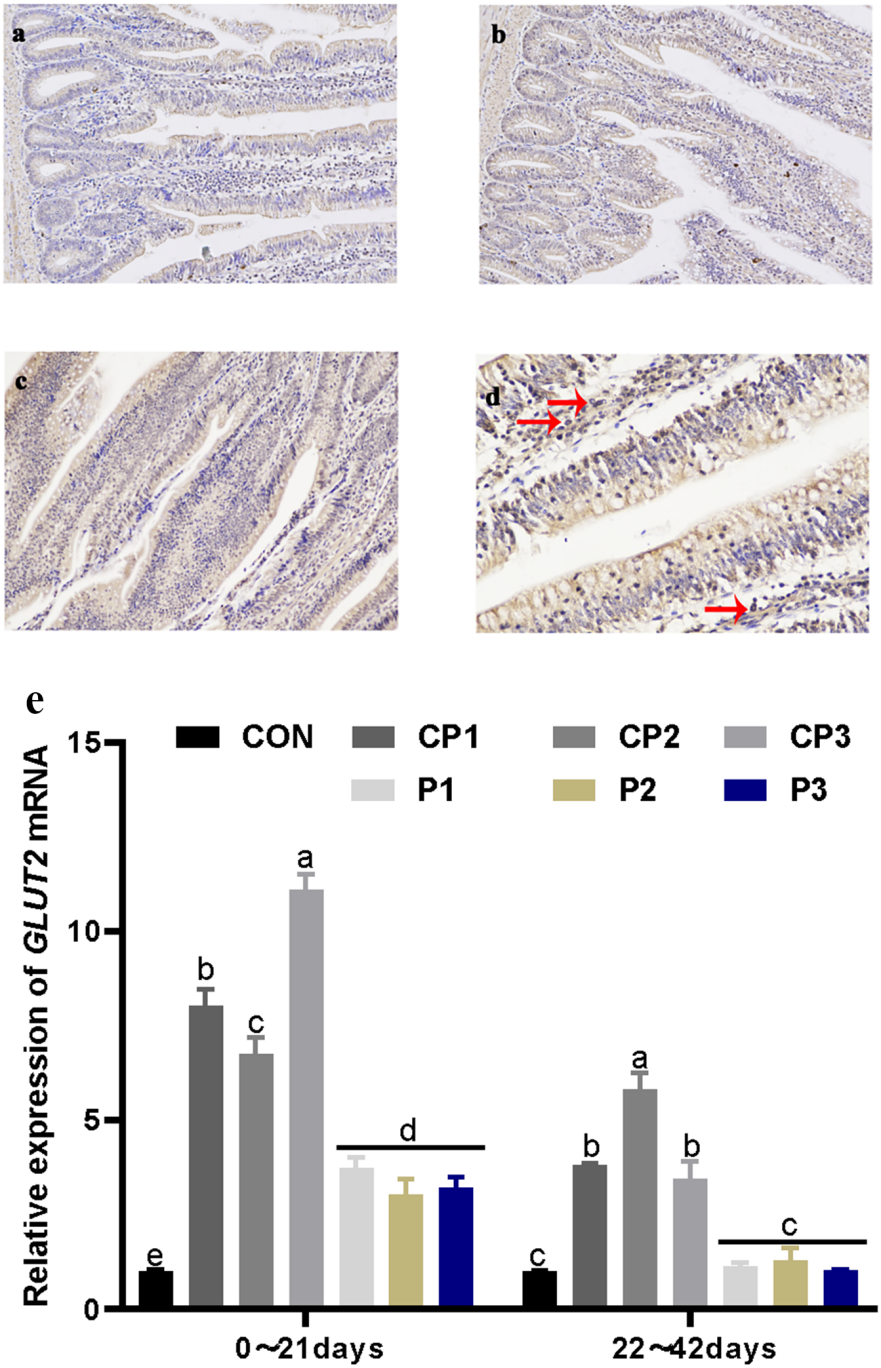
Localization and expression of GLUT2 in the jejunum. The staining results of jejunum GLUT2 in the control, CP and P groups, 200 × (A, B, and C). The nucleus is stained blue by hematoxylin, and GLUT2 immune response factors are brown. The positive expression of GLUT2 in the jejunum, 400 × (D). The expression of GLUT2 mRNA in the jejunum (E). The control group (broilers receiving normal drinking water), groups P1, P2 and P3 (broilers receiving drinking water with 1% *Lactobacillus casei*, *Lactobacillus acidophilus* and *Bifidobacterium lactis*, respectively) and groups CP1, CP2 and CP3 (broilers receiving drinking water with a 1% compound probiotic mixture in 2:1:1, 1:2:1, 1:1:2 ratios, respectively).

GLUT2 staining in the jejunum is shown in Figs. 2A and 2C. GLUT2 expression in each group was strong. The red arrow in Fig. 2D shows the formation of a tan vacuolar structure around the blue nucleus, indicating that GLUT2 was positively expressed in the membranes of small intestinal epithelial cells but not in the nuclei and cytoplasm. The effect of probiotics on jejunum GLUT2 mRNA expression is shown in Fig. 2E. At 1 to 21 days, GLUT2 mRNA expression in the probiotics group was significantly incresaed compared with that in the control group (*P* < 0.05). GLUT2 mRNA expression increased significantly in the CP groups (*P* < 0.05), and GLUT2 mRNA expression was the highest in the CP groups. There was no significant difference in expression in the P group (*P* > 0.05). GLUT2 mRNA expression in the CP groups was significantly higher than that in the P group (*P* < 0.05). At 22 to 42 days, GLUT2 mRNA expression in the CP groups was significantly increased compared with that in the control group (*P* < 0.05), while GLUT2 mRNA expression in the P groups was not significantly increased (*P* > 0.05). Among the probiotics groups, GLUT2 mRNA expression in group CP2 was the highest (*P* < 0.05). From day 1 to 42, GLUT2 mRNA expression decreased over time.

## Discussion

Production performance is an important index to evaluate whether poultry production is economically efficient. Our results showed that supplementation with single or compound probiotics had no significant effect on the slaughter performance of broilers, which was in line with the results of Panda et al. (2005) and Patel et al. (2015). The eviscerated carcass yield and half-eviscerated carcass yield of broilers were all over 60% and 75%, respectively, indicating that broilers had better meat charateristics. Additionally, 1% probiotics supplemented in drinking water did not have adverse effects on the broilers. Broilers who received water supplemented with compound probiotics containing a large proportion of *Bifidobacterium lactis* had better slaughter performance than those who received water supplemented with single probiotics or no probiotics. This may be because *Bifidobacterium lactis* strains that have strong survivability in the gastrointestinal tract can effectively prevent pathogenic bacteria colonization and stimulate the development of the intestinal structure to better absorb and utilize food nutrients (Liu et al., 2007; El-Moneim et al., 2020). Previous studies reported that compound probiotics and their fermented milk products had positive effects on the body weight and FCR of broilers (Ghasemi-Sadabadi et al., 2019; Javandel et al., 2019; Salehizadeh et al., 2019). We found the synergistic effects of compound probiotics in our study were better than the effects of single probiotics. However, Lee et al. (2010) proposed that supplementing *Bacillus subtilis* in the drinking water of broilers had no effect on the broilers’ daily gain despite supplementation with a single strain or multiple strains. In addition, the addition of a 0.1% *Lactobacillus* compound to feed did not affect the body weight, feed intake or FCR of broilers in any the growth stages (Chang et al., 2019; Makled et al., 2019). The differences in the effects of probiotics as feed additives may be due to a variety of reasons, including the viability of probiotics species, different probbiotic densities, different probiotics delivery methods or dosages, broilers selection, different growth environments, etc.

Increased digestive enzyme activity contributes to improved feed utilization and availability of enteral nutrition, and digestive enzymes are regarded as reliable indicators of individual nutritional status. For years, the role of probiotics in the activity of intestinal digestive enzymes has been debated. Studies have shown that *Lactobacillus* (Jin et al., 2000; Jazi et al., 2018), *Saccharomyces boulardii* (Rajput et al., 2013; Sun et al., 2017) and *Bacillus* (Gong et al., 2018) can stimulate the activities of digestive enzymes in the small intestine, but studies have also shown that *Lactobacillus* inhibits the activity of digestive enzymes (Li et al., 2015). Rodjan et al. (2018) found that single and compound *Bacillus* species had no effects on specific activities of digestive enzymes. In this study, the different stimulating effects of 1% probiotics on jejunal digestive enzyme activity were related to feeding stage and strain type. For example, probiotics containing *Bifidobacterium lactis* stimulated the activities of jejunal AMY, LPS and TPS. *Lactobacillus casei* stimulated the activities of LPS and TPS, while *Lactobacillus acidophilus* stimulated the activity of TPS from 1 to 21 days. Rapid colonization in the intestinal wall and the production of active substances can inhibit the growth of harmful bacteria, protect the intestinal tract from infection and provide a better environment for enzyme survival (Zhang et al., 2016).

The villus and crypt are special structures of the small intestine epithelium, which is composed of functionally active and constantly renewing cell groups. High villi and shallow crypts indicate a large number of mature intestinal epithelial cells, resulting in strong intestinal digestion and absorption functions (Broom, 2015; Fernandez-Alarcon et al., 2017). In this study, villus height and crypt depth in the small intestine were significantly different among each group, and single probiotics and compound probiotics had positive effects on these morphological parameters. However, the positive effects of the compound probiotics was more significant than those of the single probiotics, in accordance with previous study results (Salim et al., 2013; Shirani et al., 2019; Sokale et al., 2019; Nii et al., 2020). Studies have found that a large number of SCFAs produced during probiotic metabolism provide energy for the growth of intestinal epithelial cells and stimulate the development of intestinal mucosa, increase the surface area for intestinal absorption, and improve the efficiency of feed utilization in broilers (Wang et al., 2017; Biasato et al., 2018). This may contribute to the reason why probiotics stimulate digestive enzyme activity and increase ADG in broilers. Song et al. (2014) found that compound probiotics could increased the abundance of *Lactobacillus* and *Bifidobacterium* in the small intestine and improved the morphology and structure of the jejunum. The improvements of intestinal performance parameters may be due to the ability of probiotics to inhibit pathogen growth, balance the intestinal flora, improve intestinal function, and reduce damage to the mucosal barrier (Song, li Li & Hu, 2013; Jazi et al., 2017; Singh et al., 2019). In addition, probiotics can improve intestinal morphology due to their antioxidant activity. They can inhibit the production of oxygen free radicals, enhance antioxidant capacity, and prevent oxidative damage to intestinal villi (Bai et al., 2018).

The nutrient transporter GLUT2 enables nutrients in food to be effectively absorbed by the intestines and other tissues to meet the nutrient requirements for broiler growth and maintain glucose homeostasis (Thorens, 2015). In this study, the expression of GLUT2 mRNA in the jejunum of broilers treated with probiotics was significantly upregulated. In line with this, Jahromi et al. (2016) found that probiotics increased the expression of sugar transporter genes. Woting et al. (2014) also found that *Clostridium ramosum* had this effect in the jejunum of mice. This study showed that the upregulation of the GLUT2 gene was related to probiotic type and feeding time. For example, probiotics upregulated the expression of GLUT2 mRNA; however upregulation was observed from only day 1 to 21 for single probiotics. Upregulation by probiotics may be related to total SCFAs (including propionate and butyrate) produced during fermentation. Previous studies have found that SCFAs can increase GLUT2 abundance in the gut (Nedjadi et al., 2014). Mangian & Tappenden (2009) proposed that butyrate activates the GLUT2 promoter. Age, breed, and the composition of nutrients in feed may affect the expression of nutritional transporters in the intestine (Awad, Ghareeb & Zentek, 2014; Kaminski & Wong, 2018; Hayat et al., 2020).

## Conclusions

In this study, the three probiotics and their interactions promoted the small intestinal villus development and stimulated digestive enzyme secretion in the jejunum, which improved intestinal digestion and absorption. Probiotic supplementation also upregulated GLUT2 expression in the extracellular membrane, promoting nutrient transport. The growth performance of broilers was improved. In addition, among the three probiotics, *Bifidobacterium lactis* had the highest activity in the intestinal tract, and compound probiotics improved the production efficiency of broilers more than single probiotics. However, according to the current research results, the specific mechanism regulating the effects of probiotics on nutrient transporters is still unclear, and further research is needed.

##  Supplemental Information

10.7717/peerj.13308/supp-1 Supplemental Information 1 Checklist

10.7717/peerj.13308/supp-2 Supplemental Information 2 Raw data The data was analyzed by SPSS 24.0 software and the data type was Mean ± SD.

## Additional Information and Declarations

### Data Availability

The following information was supplied regarding data availability: The raw measurements are available in the Supplementary Files.
